# Survival of fossils under extreme shocks induced by hypervelocity impacts

**DOI:** 10.1098/rsta.2013.0190

**Published:** 2014-08-28

**Authors:** M. J. Burchell, K. H. McDermott, M. C. Price, L. J. Yolland

**Affiliations:** Centre for Astrophysics and Planetary Science, School of Physical Sciences, Ingram Building, University of Kent, Canterbury, Kent CT2 7NH, UK

**Keywords:** diatoms, shock, hypervelocity, fossils, meteorites, Panspermia

## Abstract

Experimental data are shown for survival of fossilized diatoms undergoing shocks in the GPa range. The results were obtained from hypervelocity impact experiments which fired fossilized diatoms frozen in ice into water targets. After the shots, the material recovered from the target water was inspected for diatom fossils. Nine shots were carried out, at speeds from 0.388 to 5.34 km s^−1^, corresponding to mean peak pressures of 0.2–19 GPa. In all cases, fragmented fossilized diatoms were recovered, but both the mean and the maximum fragment size decreased with increasing impact speed and hence peak pressure. Examples of intact diatoms were found after the impacts, even in some of the higher speed shots, but their frequency and size decreased significantly at the higher speeds. This is the first demonstration that fossils can survive and be transferred from projectile to target in hypervelocity impacts, implying that it is possible that, as suggested by other authors, terrestrial rocks ejected from the Earth by giant impacts from space, and which then strike the Moon, may successfully transfer terrestrial fossils to the Moon.

## 1. Introduction

The successful transfer of biological material, or indeed complex organic molecules, around the Solar System, most likely involves an extreme shock event when the material arrives at a new body. A typical scenario has a rock (carrying the biological material) travelling through space and arriving at high speed at another body. The speeds are high because orbital mechanics dictates speeds in the range of km s^−1^ and sudden deceleration from such speeds typically involves shocks in the 1–100 GPa range. It had long been thought that shocks even in the 10 and 100 s of MPa range were sterilizing events, but in the early 2000s it was shown in laboratory impact studies that this was not the case: it was found that *Rhodococcus erythroplis* survived in impacts on nutrient agar broth at 5 km s^−1^ [1] and that *Bacillus subtilis* spores could survive shocks at 32 GPa in flyer plate experiments [2]. More work has followed since, showing how the survival rate for a variety of microorganisms varies with shock pressure in both high-speed impacts [3–5] and for shocks induced in samples via flyer plates [6,7]. The survival rates reported were at the level of 10^−4^–10^−7^, but this should be contrasted to the observation that a gram of terrestrial soil typically contains 10^8^ spores and microorganisms.

The idea that biological material can successfully migrate through space is an old one. Scientific discussion of the possibility dates back into the nineteenth century, and in the early twentieth century the Swedish scientist Arrhenius named it Panspermia [8]. The early versions of Panspermia had seeds floating freely through space driven by radiation pressure, but then, as appreciation grew of the hazards in space presented by radiation acting on biological samples, the idea of Panspermia fell into decline (see [9] for a review).

However, different versions of Panspermia subsequently emerged. In particular, litho-Panspermia became popular [10]. This posits that life starts on a planet and is present in the soil and rocks of that body. A giant impact from space then occurs and throws up high-speed ejecta which escapes into space. After a period in space, its orbit takes it into an impact with another body which can potentially serve as a new home. Key to this process are two phases involving shocks. The first is the initial launch into space, when the material is ejected. The second, more extreme shock is upon arrival at the new home. In the former case, it was shown experimentally that microbial life frozen into ice could undergo high-speed ejection after an impact into the ice and remains viable [11]. Later, it was similarly shown that bacteria on a rocky surface could survive high-speed ejection from an impact site [12]. That microbial life could survive high-speed impacts had already been established (see above), so Panspermia *per se* is no longer explicitly ruled out, although we have no evidence for life existing other than on the Earth.

This subject of extreme shocks on biological materials has grown in recent years and covers various aspects of work relating to Panspermia. The questions asked and the related scientific interest are summarized in table 1 (where we split the issue into two, relating to whether the material of interest is in the target or on the projectile). The least complex structures considered are complex organic molecules. These are precursor materials for life or indicators of the presence of life if they have a purely biological origin. Much experimental work has been done on their shock synthesis or breakdown during impacts and it would require a major review to summarize this in detail. Put briefly, it is even possible to synthesize amino acids from basic precursors [13]. Moving to life itself, survival of microbial life forms in shock events has already been discussed above. Larger biological structures than microbes offer difficulties however. Seeds may seem an obvious route for Panspermia: they could be trapped in rocks near the site of a giant impact and launched into space and eventually impact a new body and be released into a new environment. However, their complicated internal structure seems to render them prone to damage as shocks of order 1 GPa pass through them [14,15]. Therefore, at laboratory scales, they do not seem suitable vehicles for Panspermia.

**Table 1.** Scientific questions relating to Panspermia.

	complex molecules	microbial life	seeds	fossils
present in the target	impact-driven shock synthesis	extinction, or launch into space as ejecta	source of material for litho-Panspermia or re-seed a planet after an extinction event	possible launch into space, alteration of fossil record at impact sites.
carried on the impactor	exogenous delivery of complex molecules	Panspermia	litho-Panspermia or re-seed a planet after an extinction event	a record which can be preserved in space or elsewhere

But it is not just Panspermia itself that is of interest. If material were moved from one place, it could be preserved in storage at a new home. This is the suggestion of several authors who imagine giant impacts sending ejecta outward from the Earth which then hits the Moon. This ejecta may contain material of biological interest and it may be preserved for long periods in more stable conditions than if it had remained on the Earth [16–18]. That geological materials in general which impacted the Moon can survive and be recognized in lunar soil has been shown from examination of lunar samples returned by the Apollo astronauts [19]. It has also been shown (via hydrocode modelling) that approximately a quarter of all asteroids which strike the Moon do so at speeds of less than 12 km s^−1^, and that the resulting shock pressures inside these impactors are sufficiently low so as to enable survival of significant amounts of the projectile material [20]. In the case of material ejected from the Earth striking the Moon, the impact speeds are lower still, peaking at approximately 3 km s^−1^ [21]. This, in turn, leads to lower peak shock pressures (estimated in the range 1–20 GPa depending on the materials and impact speed [18]). There may thus be extensive survival of terrestrial materials on the Moon.

What is of interest in this paper is not survival of complex organic molecules or microbial life in impacts at these Earth–Moon impact speeds and shock pressures, but rather the survival of fossils. This is because while it has been suggested that fossils may be present in Earth ejecta on the Moon [16–18], no one has previously demonstrated whether they can indeed survive such impacts. Therefore, we report on experiments using a two-stage light gas gun to fire projectiles at speeds of between approximately 0.4 and 5 km s^−1^ into targets of water. We then recover the surviving projectile material from the target and examine it. The fossils we use in the projectiles are from diatoms. Diatoms are unicellular, photosynthesizing algae encased in a shell of silica, called the frustule. They readily make recognizable fossils which have been well characterized in a wide variety of previous studies. As well as being extant today, diatoms have been identified in the fossil record back to the Lower Cretaceous (approx. 140–100 Ma), and their use has been posited as a significant tool in providing records of conditions at their time of formation [22].

## 2. Material and methods

**Figure 1.**
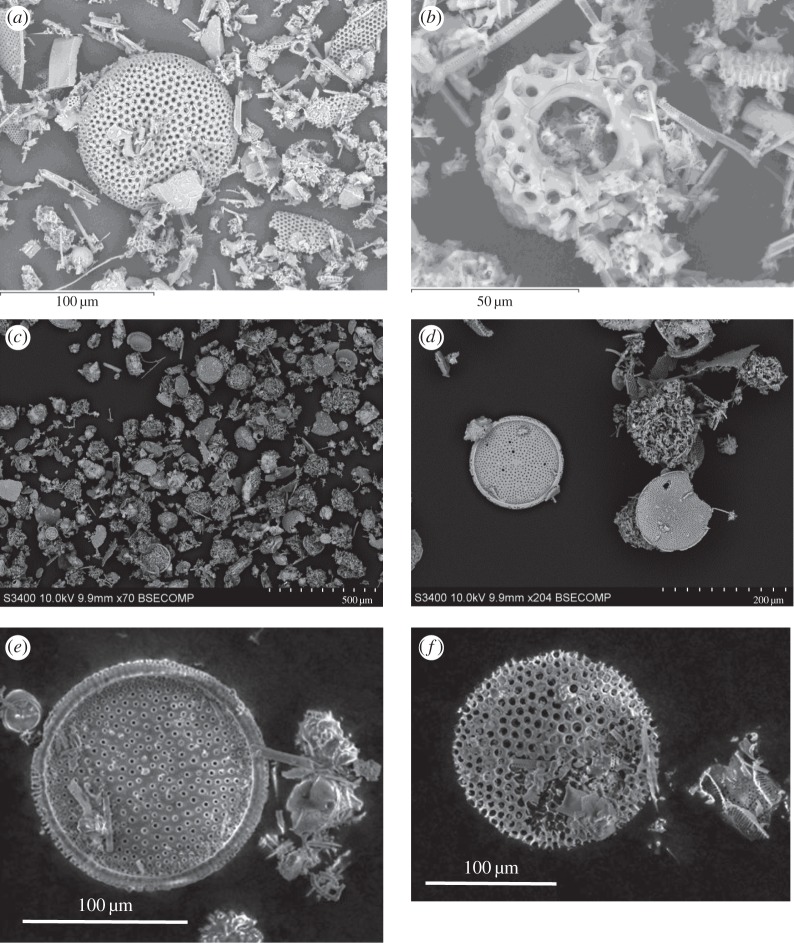
Secondary electron images of raw diatoms from diatomaceous soil pre-shot (*a*,*b*). Back-scattered electron images of (*c*,*d*) sieved fossilized diatoms pre-shot and (*e*,*f*) fossilized diatoms recovered from a frozen but un-shot projectile.

The diatom fossils used here were obtained from diatomaceous earth. This is rock which formed predominately from fossilized diatoms and here is in a powdered form. Diatoms in general, range from 2 to 2000 μm in size. Intact diatom fossils were readily apparent mixed with broken ones in this powder (sourced from Sigma-Aldrich), with a maximum size of 180 μm. Examples are shown in figure 1*a*,*b*. To prepare for being fired in the gun, the diatomaceous earth samples were poured into a cylindrical solid nylon projectile (density 1184 kg m^−3^) into which a central hollow shaft had been drilled. The projectile had a height 4.3 mm and external diameter 4.5 mm. The central shaft had an internal diameter of 3 mm and extended 3 mm into the projectile from one end face. Once the diatomaceous earth (mass 3.3 μg) had been poured into the projectile, water was then poured in to fill the space. The projectile was then frozen to −20°C, fixing the diatom material into position.

The gun used in this work was a two-stage light gas gun at the University of Kent [23]. This fires a shotgun cartridge in a first stage, which drives a piston to further compress a pre-compressed light gas in the pump tube. This gas is suddenly released from its high pressure when a containing disc of aluminium bursts. This releases the gas into the second stage (launch tube) where it accelerates the projectile (or sabot containing the projectile, depending on the shot configuration) to high speed. The projectile is then launched (horizontally) into the range of the gun. During its flight along the gun, the projectile crosses two laser light screens. The intensity of light in these screens is monitored by photodiodes. The resulting interruption in the photodiode outputs gives timing information on the flight of the projectile. The laser screens have a known separation and this, combined with the flight time, gives the speed of the projectile (accurate to within ±1%). The speed can be controlled in each shot by varying the pre-pressure of the gas in the pump tube and varying the amount of gunpowder used in the shotgun cartridge. In normal operation, the gun has a minimum speed of around 1 km s^−1^. However here, in one experiment, the containing disc between the two stages was deliberately broken at a lower than normal gas pressure to launch the projectile at a speed significantly less than 1 km s^−1^. This permitted a low-speed shot to be made in the same apparatus as the high-speed (more than 1 km s^−1^) shots, allowing a direct comparison of the results within the same set-up.

Nine shots were carried out in this work (at speeds of 0.388, 1.26, 2.05, 3.00, 3.14, 3.33, 4.10, 5.11 and 5.34 km s^−1^). In most of the work here, the projectiles contained samples of the raw diatomaceous earth used direct from the main supply. However, we also sieved the sample into four discrete size ranges (more than 75, 75–90, 90–125 and 125–180 μm). Examples of sieved fossils are shown in figure 1*c*,*d*. In the shot at 3.00 km s^−1^, just material in the size range 90–125 μm was used and in the shot at 3.14 km s^−1^ an equal mix of all four size ranges was used. In the size-sorted cases, the intent was to compare samples enriched in intact larger fossils with an unsorted sample at similar speed (3.33 km s^−1^).

**Figure 2.**
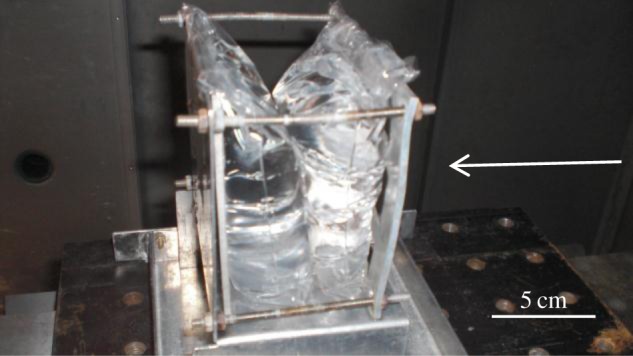
Target holder with the impact direction from the right. With the arrangement shown the target depth of water was 76 mm, only one bag of water was used in the shots where this was 37 mm. (Online version in colour.)

The target chamber was a cube with interior volume of 1.73 m^3^. Along with the range of the gun, this was evacuated to a pressure of 50 mbars during shooting; this was to avoid the projectile slowing down in flight. The target was a thin walled (approx. 6 μm thick) plastic bag of water held vertically in the target chamber (figure 2). The water was reverse osmosis purified water. In most shots, the depth of water presented along the line of flight of the projectile was 76 mm, but in the shots at 3.00 and 3.40 km s^−1^ the column water depth was 37 mm—in both cases, this was found to be sufficient to contain the impact in the water. During a shot, the target stood in a tray with a box placed over it. The box had a narrow circular opening to permit the projectile to strike the target. During the impacts, the water was forced out of the bag (which ruptured) and collected in the lower part of the bag or the tray beneath.

After the shot, two methods were used to prepare the samples for analysis. In almost all of the shots, the collected water was poured through Whatman filter paper to collect any particulate matter. To assist in this collection, the target was rinsed with more reverse osmosis purified water and this was also poured through the filter paper. The paper was then dried and its contents imaged. However, as a test of the effectiveness of this method, in the shot at 3.33 km s^−1^, a sedimentation method was used whereby the sample and target water was collected into a conical flask and left for a day for the solid material to settle at the base. The excess water was removed using a glass pipette and placed into a second flask until a small volume remained at the base with the solid projectile sample. The sample and remaining water were transferred to 15 ml centrifuge tubes and the sedimentation and removal of excess water was repeated until only a small volume of water remained mixed with the solid sample material. The final stage of this process allowed the water to evaporate by placing the centrifuge tubes into an oven set at 90°C for 2 h. Once dried, the solid sample material was collected and imaged. All excess water was kept and checks were undertaken to ensure that no solid material had been mistakenly neglected; if it had the method was repeated. Both the filtration and sedimentation methods were able to separate the solid residue from the water for analysis with no apparent difference between their effectiveness. Clean glassware, previously un-used with diatoms was used in each shot.

The images obtained in this work were made with a scanning electron microscope (model Hitachi S3400N). This was also equipped with an EDX system (Oxford Instruments ‘Xmax-80’ silicon drift detector and ‘Inca’ software, calibrated using a cobalt standard) permitting elemental analysis. The recovered material from each shot was imaged in the scanning electron microscope (SEM) and searched for examples of intact diatoms. As well as being used to search for intact diatom fossils, the SEM was used to image large amounts of material in selected shots and the size of the fossils (or fragments) measured. This was done in six of the nine shots (where multiple shots were done at similar speed, i.e. 3 and 5 km s^−1^, only one shot at each speed was analysed).

Two tests were carried out of the analysis method. In the first, a volume of diatomaceous earth similar to that used in a shot was added to a small amount of water. This was then filtered using the first of the two analysis methods and the resulting filter paper examined. Intact diatom fossils were readily recognized in the SEM images, indicating the analysis method could extract diatom fossils. As a further test, a frozen projectile containing fossilized diatoms was placed directly (without being shot) into a volume of liquid water similar to that used as a target. This water was then handled as if it were from a target post-shot and prepared for analysis via the sedimentation method. The result was again that diatom fossils were readily identifiable in the SEM, including large (more than or equal to 100 μm) fossils (figure 1*e*,*f*). This provides evidence that freezing had little, to no effect, on the physical structure of the diatom material.

## 3. Results

At the lowest speed (0.388 km s^−1^), we found copious survival of fossilized diatoms and the mean size was similar to that in the control sample which had not been shot in the gun (table 2). There was little in the way of gun or target-related debris in the sample so it was relatively easy to study the surviving fossilized diatom material in the SEM.

**Table 2.** Size of fossil fragments as recorded from SEM images.

shot velocity (km s^−1^)	total fragments	average size (μm)	largest fragment size (μm)	second largest fragment size (μm)
un-shot control	500	28.8±0.8	180	136
0.388	500	32.6±0.8	119	105
1.26	126	20.0±1.1	80.7	62.8
2.05	81	19.1±1.1	58.9	45.8
3.14	33	15.9±1.5	40.2	39.7
4.1	31	17.3±1.6	39.6	36.8
5.11	17	16.9±3.7	61.8	46.9

In the shots at speeds above 1 km s^−1^, there was an increasing amount of debris in the recovered samples. This included bits of broken sabot and torn plastic bag. The fossil fragments were mixed with this debris making their measurement harder. In addition, at the higher speeds there were fewer fossil fragments readily visible. In none of the shots above 1 km s^−1^ did we find large intact diatom fossils although small intact fossils were found. For example, in the shots at 1.26 and 2.05 km s^−1^, we saw intact fossils of sizes 17–40 μm.

**Figure 3.**
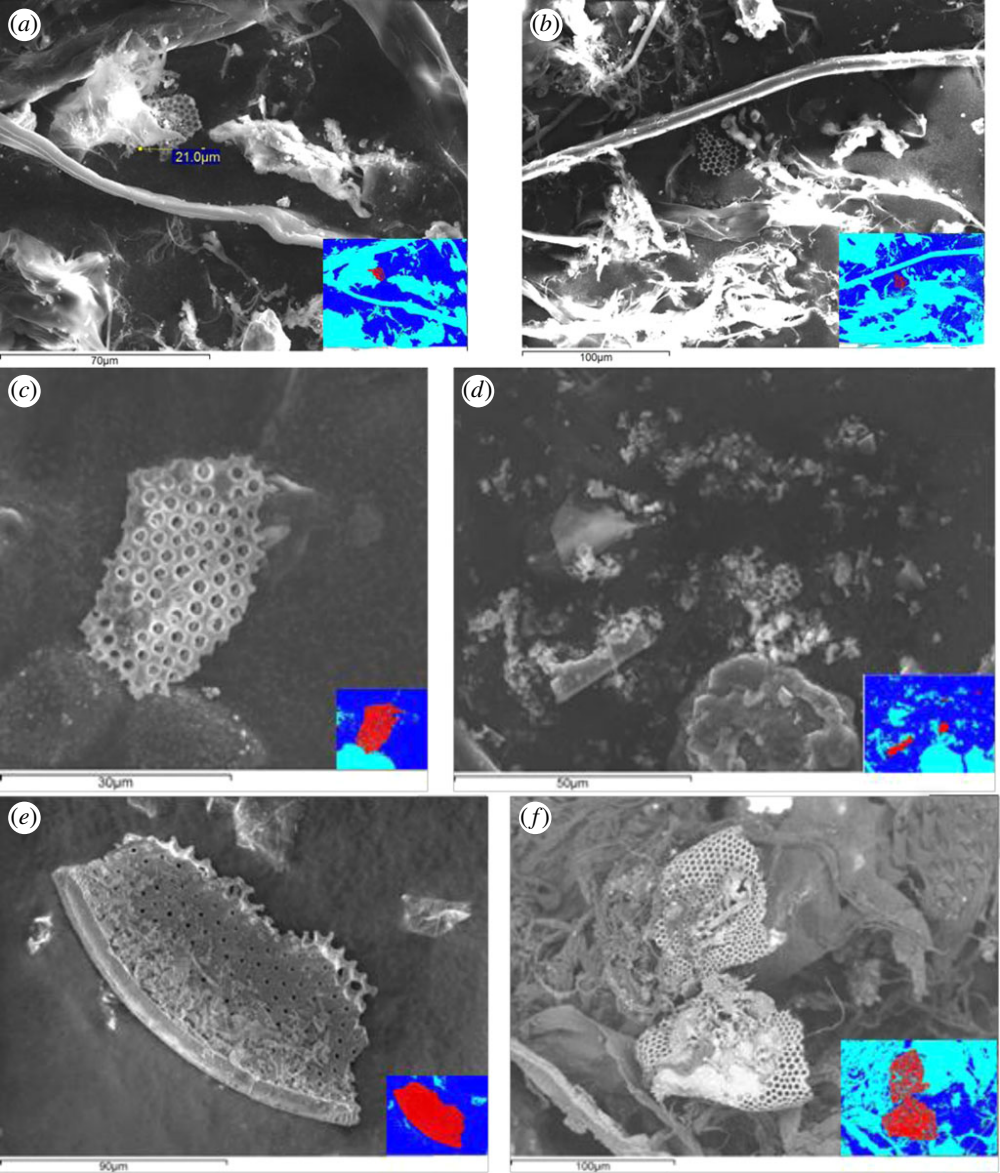
Secondary electron SEM images of typical fossilized diatom fragments trapped in debris from the shots at ≈3 km s^−1^ impact speed. To aid identification, the insert (bottom left in each image) shows the fossilized diatom coloured red (appears as grey in print version), the carbon stub coloured dark blue and gun debris coloured light blue. (*a*,*b*) Results from shot at 3.14 km s^−1^. In (*a*), the fragment is 21 μm, whereas in (*b*) it is ≈40 μm across. (*c*,*d*) Results from shot at 3.00 km s^−1^ using seived diatom material between 90 and 125 μm. Several fragments are visible, ranging in size up to about 30 μm. (*e*,*f*) Results from shot at 3.33 km s^−1^. The fragments in (*e*,*f*) are about 100 μm. (Online version in colour.)

In the shots at 3 and 4 km s^−1^, we saw no intact fossils at all. The shot at 3.14 km s^−1^ was typical. Analysis of the recovered material showed many examples of fossilized diatom fragments, at sizes up to 40 μm across (figure 3*a*,*b*). However, no intact fossils were found. As already stated, some of the recovered material was mixed with debris from the shot itself or parts of the plastic bag the target water was in, so not all the material could be fully examined. Thus, the search for intact fossils was not exhaustive, but we can say that their frequency was notably reduced from that in the original sample. We also studied the SEM images from the shot at 3.00 km s^−1^ where the projectile contained only sieved diatom material between 90 and 125 μm. This was chosen because of all the sieved material this size range contained the greatest volume of intact whole diatoms. The analysis of the captured material in the SEM again did not yield any examples of intact diatoms. However, many examples of diatom fossil fragments were obtained, although all were less than 50 μm in size (figure 3*c*,*d*). Similarly, the shot at 3.33 km s^−1^ yielded no intact fossils, although we did find fragments of size around 100 μm, notably bigger than in the other shots at around 3 km s^−1^ (figure 3*e*,*f*).

The shot at 4.10 km s^−1^ gave similar results to those at 3 km s^−1^, in that we saw no intact fossils but did find fragments.

**Figure 4.**
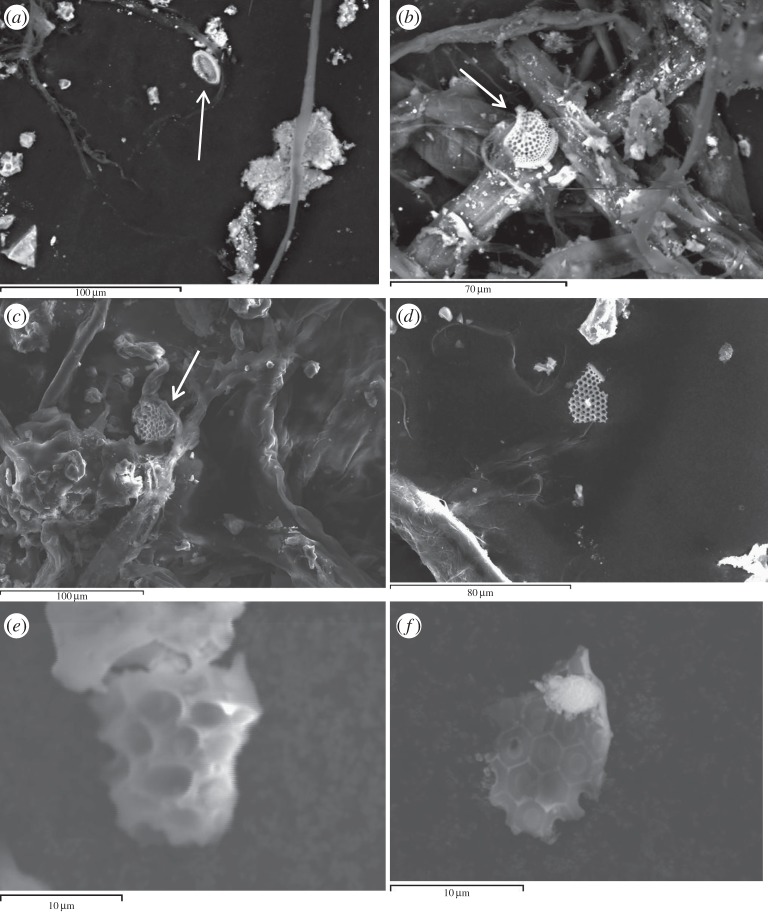
Results from shot 4 at 5.34 km s^−1^. Large area images are shown in (*a*–*c*) and include whole diatoms fossils. Close up images of smaller fragments of diatom fossils are shown in (*d*–*f*).

**Figure 5.**
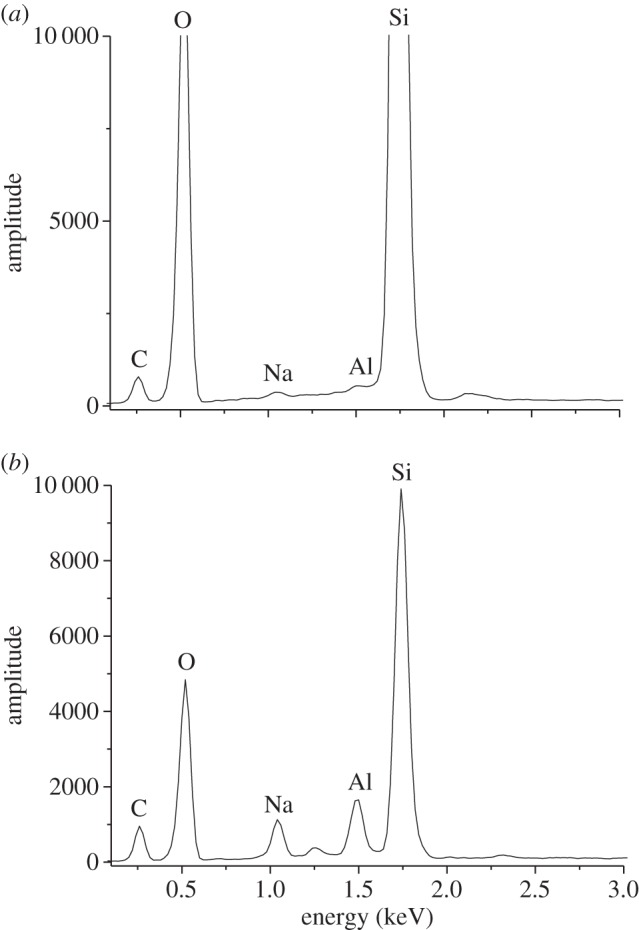
SEM-EDX spectra. (*a*) Pre-shot. (*b*) Sample recovered from shot at 5.34 km^−1^.

However, in the shot at 5.34 km s^−1^, we found four examples of intact diatom fossils, three of which are shown in figure 4*a*–*c*, broken fragments in the same shot are shown in figure 4*d*,*f*. The intact whole diatoms were all less than 30 μm in diameter in this shot. In figure 5, we show a typical SEM-EDX spectrum for a diatom fossil recovered after the impact in the water in the shot at 5.34 km s^−1^. The spectrum is directly comparable to those before impact. This adds elemental evidence to the visual evidence that we have recovered diatom fossils.

**Figure 6.**
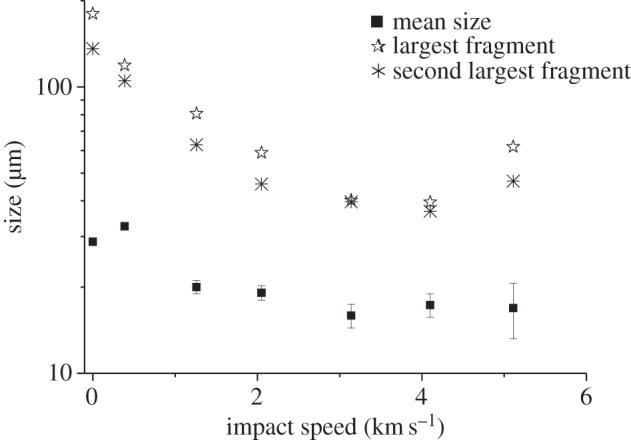
Size distribution of diatom fragments versus impact speed. The sample at 0 km s^−1^ is the un-shot, control sample. The mean size in each sample is shown, along with the largest fragment observed (which in the control sample is an intact diatom) and, for completeness, the size of the second largest fragment is also shown (see text for discussion).

As well as searching for intact fossils, analysis was made of the size of the fragments that were recovered in six of the nine shots (this uses one shot at each speed, ignoring the multiple shots at approx. 3 and 5 km s^−1^). This involved scanning large volumes of the recovered material. Searching for the endpoint of a size distribution is a process which can be subject to bias (have you found the largest specimen? Is the actual largest specimen in a sample representative if the process was repeated?). Accordingly, to check the consistency of the results we also report the size of the 2nd largest fragment found. The results are given in table 2 and shown on figure 6. We found that the mean size of the fragments dropped above 1 km s^−1^, falling to approximately 20 μm at 1 and 2 km s^−1^ and falling to approximately 17 μm at speeds of 3 km s^−1^ and upwards. The largest fragment size also fell (figure 6). The largest fragments tended to be around 40 μm at the higher speeds (although slightly higher values were found at 5 km s^−1^). This limit is below the size of the majority of the intact fossils in the control sample, indicating that the majority of fossils will have been broken.

### (a) Modelling

To investigate the relation between impact speed and shock pressure, we used two methods to estimate the peak shock pressures involved in the shots. The first was the planar impact approximation (PIA) [24]. This calculates peak shock pressures in hypervelocity impacts using the linear shock wave speed
3.1
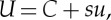

where *U* and *u* are the shock wave and particle speeds (m s^−1^), respectively, and *C* (m s^−1^) and *s* are material-specific constants which have to be known for both projectile and target materials and are normally derived from dedicated flyer plate experiments. The PIA assumes impacts between two semi-infinite bodies and finds the peak pressure just behind the contact plane. A difficulty in the experimental set-up here is that this method does not allow for a mixed material projectile. Accordingly, we calculate the peak shock pressures assuming it was water ice (which is the material the fossilized diatoms are carried in in the centre of the projectile). The results (including the *C* and *s* values used) are given in table 3.

**Table 3.** Estimates of peak shock pressures in the impacts reported here. The *s* and *C* values (equation (3.1)) for the projectile were 1.28 and 1560 m s^−1^, respectively, for water ice, and for the water target were 1.48 and 1600 m s^−1^, respectively [24].

		hydrocode shock pressures (GPa)		
impact speed (km s^−1^)	PIA peak pressure (GPa)	lowest peak pressure anywhere in the frozen sample	lowest peak pressure across most highly shocked 50% of frozen sample	greatest peak pressure anywhere in frozen sample
0.388	0.3	0.12	0.17	0.55
1.26	1.5	2.04	2.31	3.19
2.05	2.9	4.40	5.97	6.26
3.14	6.3	8.45	9.91	10.8
4.10	8.6	12.2	13.8	15.5
5.11	12.4	17.0	18.8	21.2

**Figure 7.**
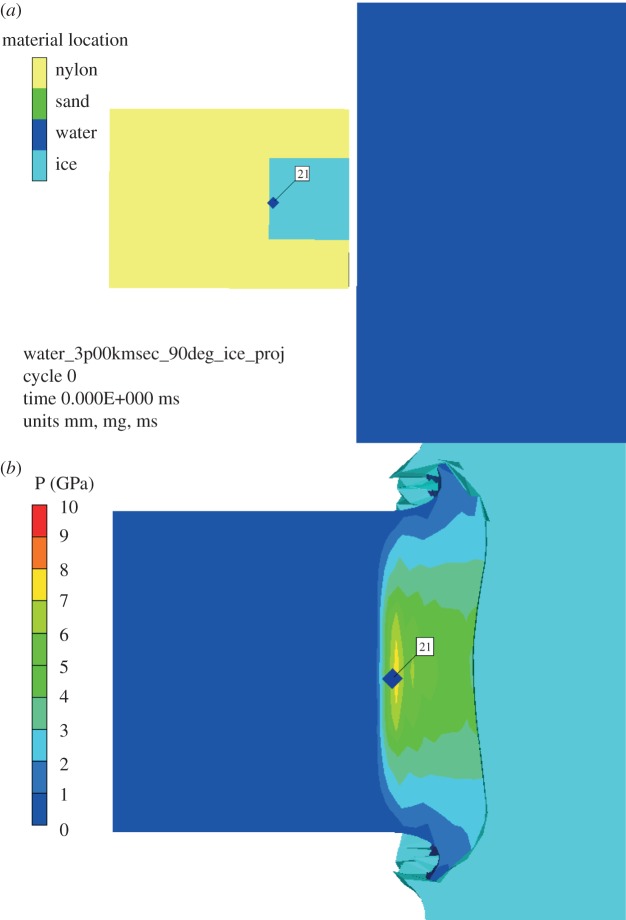
(*a*) AUTODYN model set-up of an impact at 3 km s^−1^ of a projectile containing ice striking water, showing the position of gauge 21 which experienced the highest peak pressure. The location is almost, but not quite, at the back of the ice and diatom filled cavity within the hollowed out nylon projectile. (*b*) Contour map showing the pressure distribution in the same simulation as (*a*) at the instant the peak pressure is reached. Gauge 21 (labelled) reaches a peak pressure of 10 GPa. (Online version in colour.)

**Figure 8.**
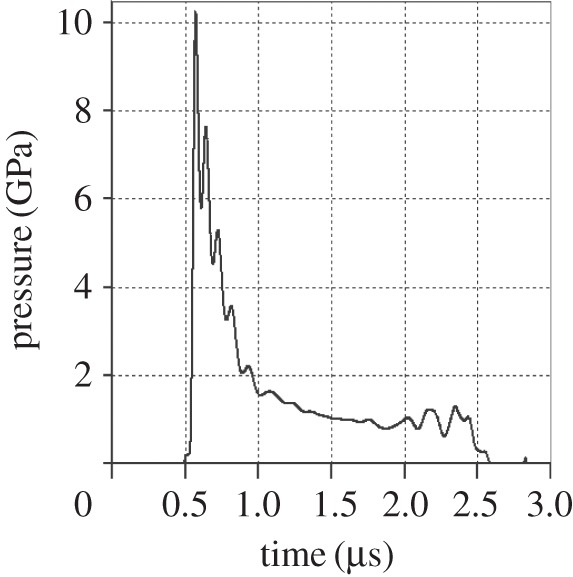
Shock pressure history of gauge number 21 (the gauge which showed the highest peak pressure in figure 7) in the hydrocode simulation of an impact at 3 km s^−1^. Note that the peak pressure is only maintained for a very short period of time (a few nanoseconds) before relaxing to approximately 1 GPa after 1 μs, then to zero after a further microsecond.

Another issue regarding the peak shock pressure is the finite extent of the projectile—the peak shock pressure will vary with depth inside the projectile. Accordingly, a hydrocode simulation of the impacts was set up. This used ANSYS' AUTODYN hydrocode package [25] and modelled the nylon projectile containing ice (e.g. figure 7). Gauge points were placed in the ice sample to record the variation of shock pressure with time at 21 discrete places in the sample. The pressures vary with time and the time evolution of the pressure at one gauge point in the 3 km s^−1^ impact speed simulation is shown in figure 8. The maximum value of the pressure was found at the rear of the ice and typically held for a timescale of 2 ns, before decaying to ≈20% of the peak value over timescales of 0.5 μs. However, the peak shock value was confined to a limited region (the other gauge points showed lower peak pressures). It is necessary to understand this variation of peak shock pressure across the projectile, as it is not known where the surviving diatoms were located in the projectile.

The hydrocode simulations showed that the design of the projectile (a nylon cylinder with a smaller ice insert at its front face) meant that the ice part was shocked to a near uniform peak pressure across its bulk, but that there were some variations in limited regions. Thus, for the impact at 3 km s^−1^ impact (shown as a typical example in figure 8), the ice sample will have experienced at least 8 GPa peak shock pressure (minimum recorded anywhere in the ice in the simulation and which was at the edges of the ice), the bulk of the ice was shocked to around 9 GPa, and, in a limited region, the maximum pressure reached was 11 GPa (table 3).

**Figure 9.**
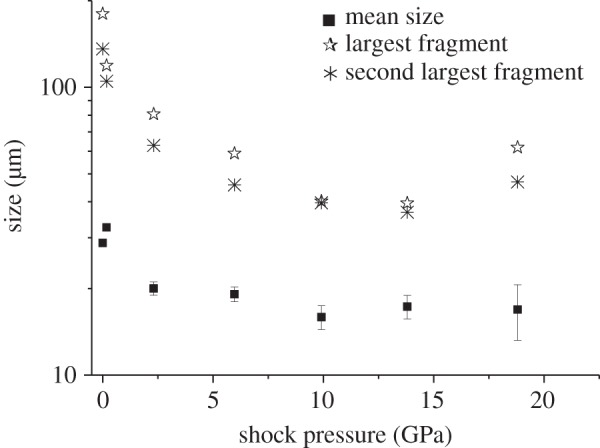
Size distribution of diatom fragments versus typical peak shock pressure in the sample. The samples at 0 GPa is the un-shot, control sample. The mean size in each sample is shown, along with the largest fragment observed (which in the control sample is an intact diatom), and for completeness, the size of the second largest fragment is also shown (see text for discussion).

In the sample configuration used here, the PIA gives a shock pressure about 75% of the minimum shock pressure experienced in the ice (at the edges of the ice where it is in contact with the sabot walls) for pressures above 1 GPa and is therefore not a good indicator of the mean behaviour of the sample. Similarly, the absolute maximum pressure in the hydrocode simulations is in a limited region and is also thus not representative of the shock history of the sample as a whole. We therefore use the lowest peak pressure in the most highly shocked 50% of the ice as a measure of what the fossil material typically experienced in a given impact. This helps provide a picture of the pressure experienced. The presence of the fossilized diatoms in the ice in the real experiments will have changed the results somewhat, but we take the values obtained in the simulations as being typical of those the diatoms would have experienced. Accordingly, we re-plot figure 6 with the *x*-axis showing the typical peak shock pressure instead of impact speed (figure 9).

From table 3 and figure 9, we can see that shock pressures of up to around 2.4 GPa throughout the sample do not reduce the mean size of the fossil fragments. However, at 2.4 GPa, the fragment size is reduced and this falls again above 6 GPa. In addition, the largest fragment size also falls at these shock pressures.

## 4. Conclusion

We have shown for the first time that it is possible to recover whole diatom fossils fired at a speed of 5.34 km s^−1^ into water targets with typical peak shock pressures of 19 GPa (the lowest peak shock pressure in the sample at this speed was 17 GPa). In addition, in all shots, we readily found fragments of fossil diatoms although the mean fragment size decreased with impact speeds above 2 km s^−1^ and shock pressures of 2.3 GPa. The fossils were recognized by their distinct visual appearance as well as by elemental analysis. In our work, the intact fossils were not as readily apparent as in the control samples that were analysed, indicating that many of the originally intact fossils broke during the shock event.

We did not find any intact large diatoms in any shot above 1 km s^−1^; although intact fossils were found, their maximum size was 40 μm at intermediate speeds and 30 μm at 5 km s^−1^. This is associated with the reduction in mean size of the fossil fragments, implying that the larger structures are broken first at the lower shock pressures.

The work complements other laboratory impact studies which show that fossilized biomarkers can survive impacts at similar speeds [26,27]. There is thus a growing body of work that demonstrates that material of interest regarding the origin and distribution of life in the Solar System can survive impacts. As stated earlier, it has previously been suggested that the Moon is a good place to look for terrestrial meteorites which contain fossils [16–18] and here we show that this is indeed viable.

There have also been reports that meteorites found here on the Earth contain putative fossils (e.g. from Mars, see [28]). In the case of Martian meteorites, the report of fossils sparked a major debate, which focused on whether the observed structures were really fossils or had formed via geochemical means alone—with the geochemical interpretation being the dominant consensus. Subsequent debates have also focused on whether fossils could form on Mars as on the Earth [29] and how to recognize fossils in ancient settings [30]. It however seems to have been accepted that once formed, fossils would survive the shocks involved in the high-speed impact events associated with their distribution across space. The value of the work reported here is that this is now demonstrated, although we note that there is a shock-pressure-related size effect in terms of intact survival, with structures greater than 100 μm unable to survive at shock pressures above 2.3 GPa.
